# The Structure, Diseases, and Treatment of the Teeth Considered, with a View to the Abolition, in All Common Cases, of the Pernicious Practice of Tooth Drawing

**Published:** 1842-06

**Authors:** 


***The Structure, Diseases, and Treatment of the Teeth considered, with a view to the abolition, in all common cases, of the pernicious practice of Tooth Drawing.*** **By WILLIAM WARDROPER, M. R. C. S. S. &c. LONDON, 1838, pp. 59, 8vo.**

The object of the author of this work, seems rather to have been to increase the repugnance which most persons feel to having a tooth extracted, than to impart any really useful or valuable information. The practice recommended a few years since by Mr. Fay, of London, of excising the crowns of such decayed teeth as cannot be restored by proper surgical treatment, in place of removing them altogether from their sockets, and which so soon sunk into merited disrepute, is that which Mr. Wardroper, and apparently unconscious that it had ever before been advised, proposes to substitute for extraction. Opposed as this practice is, to every thing like reason and sound philosophy, it never had many advocates, and yet had Mr. Wardroper been aware, at the time he wrote his little treatise, of the deplorable examples of injury that had resulted from it, he probably would not have become its champion. We could enumerate a number of cases, were it necessary, of protracted suffering occasioned by it that have fallen under our own observation, but the pernicious tendency of this unscientific practice, is too well known to need comment.

It would be well for Mr. W. before he again attempts to inveigh a practice which both reason and experience teach to be correct, to make himself better acquainted with the laws of the animal economy, than the views set forth in his present treatise, evidences him to be. We have no doubt, however, that his own observations and experience, have, long before this, convinced him of the incorrectness of the opinions which he has advanced.—*Balt. Ed.*

